# Can the One Health Approach Save Us from the Emergence and Reemergence of Infectious Pathogens in the Era of Climate Change: Implications for Antimicrobial Resistance?

**DOI:** 10.3390/antibiotics9090599

**Published:** 2020-09-14

**Authors:** Smitha Gudipati, Marcus Zervos, Erica Herc

**Affiliations:** Department of Infectious Disease, Henry Ford Hospital, Detroit, MI 48202, USA; mzervos1@hfhs.org (M.Z.); Eherc1@hfhs.org (E.H.)

**Keywords:** climate change, One Health, *Candida auris*, COVID 19, emerging pathogens, antimicrobial resistance

## Abstract

Climate change has become a controversial topic in today’s media despite decades of warnings from climate scientists and has influenced human health significantly with the increasing prevalence of infectious pathogens and contribution to antimicrobial resistance. Elevated temperatures lead to rising sea and carbon dioxide levels, changing environments and interactions between humans and other species. These changes have led to the emergence and reemergence of infectious pathogens that have already developed significant antimicrobial resistance. Although these new infectious pathogens are alarming, we can still reduce the burden of infectious diseases in the era of climate change if we focus on One Health strategies. This approach aims at the simultaneous protection of humans, animals and environment from climate change and antimicrobial impacts. Once these relationships are better understood, these models can be created, but the support of our legislative and health system partnerships are critical to helping with strengthening education and awareness.

Climate change has become a controversial topic in today’s media despite decades of warnings from climate scientists [1,2]. Reports of “The Hottest Month Ever Recorded,” “Glaciers Melting” and “Earth’s Food Supply Under Threat” are news headlines with which people have become familiar [3]. Most recently, the Australian wildfires are another example of the urgency to address climate change. Additionally, several studies suggest climate change is contributing to infectious disease emergence [4]. The Centers for Disease Control and Prevention stated that the impact of climate change on human health is significant [5]. Increasing temperatures have led to extreme weather resulting in rising sea and carbon dioxide levels, which contributes to increased prevalence of infectious agents. These changes affect adaptations in vector ecology, water quality and decreased nutritional supply that are associated with infectious diseases such as tickborne encephalitis, cryptosporidiosis and leptospirosis [2,6]. It also contributes to increased antimicrobial resistance [7]. We analyze the One Health approach, which focuses on the simultaneous protection of humans, animals and the environment from climate change and antimicrobial impact [8].

The relationship between global warming and infectious pathogens dates back many years. For example, Roman aristocrats used to vacation in the summer at hill resorts to avoid malaria [9]. Although humans thought they could “outsmart” these infections, many viruses, bacteria, protozoa and multicellular parasites have evolved to the human species as their natural reservoir through vector-borne transmission [10]. These infectious agents adapt to their optimal climate that includes temperature, precipitation, elevation and daylight duration. According to the Centers for Disease Control and Prevention, about 75% of emerging diseases and 60% of known human infectious diseases originate in animals. It is imperative that we learn to diagnose and control zoonotic infections, while evaluating the impact of human activity and environmental change on nonhuman reservoirs of disease [11].

Even though climate change impact and its effect on infectious diseases are well reported, it is occurring at an accelerated rate. Legionnaires’ disease, a waterborne illness, is rising with a rate of reported cases increasing 5.5 times from 2000 to 2017 [12]. Vector-borne infections such as Zika, Chagas disease, dengue and chikungunya that are usually localized to tropical climates as they require higher temperatures to complete their life cycle, are slowly migrating towards temperate climates, such as the United States, as global temperatures are increasing even in winter months [13]. Malaria is the most prevalent vector-borne disease globally [14,15]. Temperature and humidity are among the most important factors for disease transmission and extrinsic incubation period, and are facilitating the spread of malaria into areas that are currently malaria-free large urban highland populations [9]. Similarly, dengue viruses are traditionally transmitted in the tropics because frost and sustained cold weather kills adult mosquitoes and overwintering eggs and larvae [16,17]. Warming trends are shifting the vector and disease distribution to higher latitudes and altitudes. Warmer temperatures reduce the larval size of the *Aeagypti* mosquito as well, requiring the adult to feed more frequently, increasing bite rates and spread of infection [18,19]. Additionally, mosquito-borne diseases, such as West Nile virus, that typically occur during the rainy season are now occurring during the drought season. This shift is occurring because mosquitoes are brought into proximity with birds at scarce water sources, enhancing the transmission of the virus in the enzootic cycle [13,20,21].

Tickborne infections that were once thought to be confined to the Northeast United States are now expanding throughout the Midwest and further West. This is in part due to increased temperatures, which are increasing the survival and activity period of ticks, allowing extension of the range of both the reservoir and tick hosts. Additionally, this prolongs the duration of the season when people are exposed to the ticks [22]. Bacterial and protozoan tickborne diseases doubled in the United States between 2004 and 2016 as reports of both *Ixodes scapularis* and *Amblyomma americanum* ticks are expanding to new areas [23,24]. These ticks are known to transmit infections such as Lyme disease, *Anaplasma phagocytophilum*, babesiosis, human monotrophic ehrlichiosis and tularemia.

Diarrheal diseases such as *Campylobacter*, *Salmonella* and cholera survive better in warmer temperatures and, with the increasing temperatures in our water systems, we have seen the reemergence of these diarrheal infections in recent years [20,25]. This is particularly alarming as we already have significant antimicrobial resistance to these Gram-negative pathogens, and as these bacteria evolve, treatment options may be limited [20,26,27,28]. For example, there have already been numerous outbreaks of *Salmonella typhi* with resistance to several key therapeutic antimicrobials such as ciprofloxacin; however, now nontyphoidal salmonellas, which are being frequently transmitted by means of contaminated water supply, have developed decreased susceptibility to fluoroquinolone antimicrobials in developing countries [29]. Additionally, Gonzalez et al. found that *Campylobacter jejuni* survives in well water for long periods of time and the effect of ciprofloxacin resistance was temperature-dependent as the resistance mechanisms in vitro increased as temperatures went from 4 to 25 °C [30].

A well-known infection that is hypothesized to have arisen from climate change is *Candida auris*, which previously existed as a plant saprophyte and gained thermotolerance and salinity tolerance from the effects of climate change on the wetland ecosystem [31,32]. *C. auris* was first isolated in 2009 from a human ear and since then has been associated with human disease in many countries and exhibited nonsusceptibility to antifungal agents [32]. This new thermotolerant *C. auris* was proposed to have been transplanted by birds across the globe to rural areas where humans and birds are in constant contact. Human migration likely led to the emergence of *C. auris* into urban healthcare environments in which antimicrobial resistance and infection control issues have arisen. *C. auris* is the only *Candida* species that has isolates shown to be resistant to all four classes of antifungal drugs, which has created higher risks for clinical infections and breakthrough infections during antifungal treatment and prophylaxis [33]. These infections have the potential to result in significant morbidity and mortality and could be on the rise as climate change continues [3].

SARS-COV-2, the virus responsible for COVID-19, originated from the interplay between humans and animals. The likely transmission of the virus came from bats and potentially used an intermediate source, the pangolin, to transmit the virus to humans [34]. Many of the initial cases had a common exposure to the Huanan wholesale seafood market that also traded live animals. On 7 January 2020, the virus was sequenced and was found to have >95% homology with the bat coronavirus [35]. Through travel and community spread, it has become a global pandemic. Bats are especially vulnerable to climate change due to low reproductive output, ecological specialization and high trophic positions [36]. Their large surface area of noninsulated wings create significant water loss that is higher than in other small animals [37]. Thus, with climate change, increased aridity and prolonged droughts in their endemic areas bats migrate to more populated areas in search of insect prey, thereby increasing their interaction with humans, and as a result, may transmit virulent infections [37,38]. Additionally, although the prevalence of confirmed community-onset bacterial coinfections are low in patients with COVID-19, 56.6% of hospitalized patients in 38 Michigan hospitals received empiric antibiotic therapy [38]. This study and many others bring to light the urgency of antimicrobial stewardship principles to prevent antimicrobial resistance during the COVID-19 era [39].

The implications of antimicrobial resistance due to the emergence and reemergence of infectious pathogens from climate change are complex as the decreasing effectiveness of antibiotics has accelerated in recent years [40]. In 2016, a U.K government-commissioned report estimated that if no action were taken, by 2050, antimicrobial resistance would cause up to 10 million annual deaths globally, reduce gross domestic product by 2% to 3.5% and cost 100 trillion US dollars [41]. A key feature of antimicrobial resistance and climate change is that antibiotic consumption and carbon use will bring about adverse future consequences [42]. Tackling this issue has led to the establishment of a global innovation fund for both antimicrobial resistance research and investing in new drugs; however, both developments are financially competing against each other [43].

Recent evidence shows that higher temperatures are associated with higher resistance levels in common pathogens such as *Escherichia coli*, *Klebsiella pneumoniae* and *Staphylococcus aureus* [44]. In an unadjusted analysis, MacFadden et al. demonstrated an increase of 10°C across all regions in the United States was associated with increased resistance of 5.1%, 3.4% and 3.1% for *E. coli*, *K. pneumoniae* and *S. aureus* respectively [45]. This association postulates that due to horizontal gene transfer, warmer temperatures could affect the way bacteria respond to certain drug mechanisms [45]. Another recent study suggested an association between temporal climate developments and carbapenem-resistance in *Pseudomonas aeruginosa* in Europe [7]. Climate change and antimicrobial resistance are also driven by the consumption of carbon and antibiotics that can provide people with valuable short term benefits but impose long term costs [44,46]. Although both antimicrobial resistance and climate change are not wholly comparable, both issues require participation from the local, national and international organizations to help solve their challenges [47].

Additionally, animal and environmental compartments play a significant role in antimicrobial resistance [48]. There are multiple links between humans, animals and environmental compartments allowing for movement and alteration of genetic elements of bacteria and creating resistance to antimicrobials [45,49]. Industrial agriculture relies heavily on the widespread use of antimicrobials for livestock farms for therapeutics, prophylactics and controversially growth promotion [48]. Agricultural usage of antimicrobials exceeds and rivals medical usage in the United States and Europe respectively, and observational studies and surveillance reports have described antimicrobial resistance in farm animals resulting from misuse [40,48]. Small doses of antibiotics from urine, feces, manure and pharmaceutical waste are also being released into the environment through rivers, lakes and soil [50]. These sublethal doses allow for antimicrobial resistance to occur as they do not reach “cidal” concentrations [51].

With the emergence and the reemergence of these infectious pathogens, our hospital systems need to be equipped with the proper resources and education. Infection control and antimicrobial stewardship efforts will need to adapt and be prepared for these pathogens as they become more resistant to antimicrobials and cause “outbreaks” in hospital settings. Sentinel, vector, syndromic and real-time surveillance will be necessary to monitor changes related to climate change to help hospitals prepare for the emergence of new pathogens [25]. Evidence-based epidemic intelligence with early identification of infectious disease threats related to climate change is crucial to help with adaptation and preparedness for these outbreaks [25]. Microbiology laboratories will need to develop advanced capacity to identify these new organisms. As more infections are transmitted from animal reservoirs, communication between veterinarians and clinicians will be necessary in order to prevent animal transmission to humans that could lead to significant morbidity and mortality [8].

Although these new infectious pathogens and potential for increased antimicrobial resistance are alarming, we can still reduce the burden of infectious disease in the era of climate change if we focus on One Health strategies. We must acknowledge both the fragility of our healthcare systems and the healthcare’s own large carbon footprint [52]. One Health is an approach that recognizes that the health of people is closely connected to the health of animals and our shared environment. This is not a new concept, but one that is becoming important in recent years as human populations are growing and expanding into new geographic areas [8,53]. More people are now living in close contact with wild and domestic animals, both livestock and pets. Close contact with animals and their environments provides more opportunities for diseases to pass between animals and people. Additionally, our planet has experienced changes such as deforestation and intensive farming practices because of climate change. Disruptions in environmental conditions and habitats can provide new opportunities for diseases to pass to animals. Finally, the movement of people, animals and animal products has increased from international travel and trade leading to global transmission of pathogens as we are now seeing with COVID-19 [53,54,55].

The One Health concept is gaining recognition in the United States and globally as an effective way to fight health issues at the human–animal–environment interface. Preventative One Health strategies, such as mass vaccinations of animal populations can help reduce livestock-mediated zoonoses, and is both feasible and cost-effective [56]. Livestock vaccination against brucellosis and leptospirosis has been effective in reducing the burden of disease in many parts of the world where diagnosis and treatment of these diseases are limited [56,57,58]. International implementation by the World Health Organization has already utilized the One Health approach for Influenza A H1N1 in 2009, Polio in 2014, Ebola in 2014 and the Zika virus in 2016 by declaring them potential public health emergencies of international concern. This approach has had relative success by incorporating aspects of human, animal and environmental health to help with mitigation of these diseases [59]. However, there is controversy if One Health will be enough to fight climate change [8]. To implement this protection, we need to learn more about the underlying complex relationships of these pathogens and vectors and develop well-designed mitigation measures. Successful public health interventions require the cooperation of human, animal, and environmental health partners. Professionals within these sectors need to communicate, collaborate and coordinate activities [53,54]. Once these relationships are better understood, these models can be created, but the support of our legislative and health system partnerships are critical to helping with strengthening education and awareness.
